# Biliary Cripple and the Spectrum of Complications following Cholecystectomy: A Case Report

**DOI:** 10.1155/2022/5370722

**Published:** 2022-09-22

**Authors:** Anish Kumar Shrestha, Jayant Kumar Sah, Bikal Ghimire, Aashna Bhandari, Anisha Shrestha

**Affiliations:** Department of GI and General Surgery, Maharajgunj Medical Campus, Institute of Medicine, Tribhuvan University, Nepal

## Abstract

Bile duct injury is a complication seen during cholecystectomy. Here, we highlight the occurrence of bile duct injury (BDI) during an open cholecystectomy who underwent hepaticojejunostomy (HJ), later presenting with a stricture of HJ. Percutaneous transhepatic biliary drainage (PTBD) was performed which led to the development of hepatic artery injury.

## 1. Introduction

Bile duct injury is a serious complication of cholecystectomy with long-term morbidity, impaired quality of life, and reduced survival. This devastating complication can turn an individual into a “biliary cripple.” The reported incidence of bile duct injury after conventional open cholecystectomy ranges between 0.1 and 0.5 [1, 2]. Some injuries remain unrecognized for many years, only to present when patients develop secondary biliary cirrhosis. Dissection of Calot's triangle to realize the critical view of safety and identification of Rouviere's sulcus are the strategies that can be used to avoid biliary tract injuries [3, 4]. A multidisciplinary approach involving experienced hepatobiliary surgeons and interventional radiologist at a tertiary referral center is needed for proper management of BDI cases. Management of such complication aims at alleviating biliary obstruction by temporary and permanent means as needed and prevent restenosis. PTBD which is now used widely for temporary diversion of bile flow can be associated with hepatic artery injury (HAI) which can manifest as hemobilia, hemoperitoneum, or subcapsular hemorrhage. Identification of hepatic artery injury and its location is best done by using computed tomography (CT) angiography.

In this study, we encountered patients in whom biliary hemorrhage occurred after PTBD due to rupture of a pseudoaneurysm and was managed with selective transarterial coil embolization.

## 2. Case Presentation

**Figure 1:**
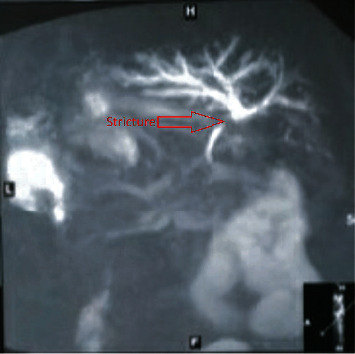
MRCP showing bismuth type II biliary stricture with tapering of bile ducts below the confluence.

A 49-year-old male underwent open cholecystectomy for acute calculous cholecystitis 8 years back. Intraoperatively, there were dense adhesions in Calot's triangle. Bile duct injury was detected and managed with T-tube placement. He was then referred to our center where he was continued on T-tube drainage and managed conservatively. T-tube was removed after 33 days, and he was kept under regular follow-up. His follow-up investigation report revealed a total bilirubin (TB) 337 umol/L, direct bilirubin (DB) 250 umol/L, serum glutamic oxaloacetate transaminase (SGOT) 56 U/L, serum glutamic pyruvic transaminase (SGPT) 65 U/L, and serum alkaline phosphatase (SAP) 1050 U/L, showing a cholestatic pattern. He was then planned for magnetic resonance cholangiopancreatography (MRCP) which revealed Bismuth type II stricture (Figure 1). For this, he was managed with PTBD and was planned for a definitive procedure. During his follow-ups, his bilirubin level gradually decreased. He underwent hepaticojejunostomy for biliary stricture after 5 months of his initial surgery, and his postoperative period was uneventful and was discharged on 5th postoperative day.

**Figure 2:**
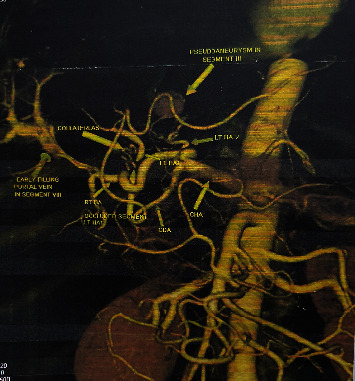
Left hepatic arteries (left HA) to segment II/III arising from proximal part of hepatic artery proper and left hepatic artery to segment IV arising from hepatic artery proper as one of its terminal branch. Contrast filled out-pouching in segment III of left lobe of liver suggestive of pseudoaneurysm.

He was on regular follow-up, and his liver function test came to normal (TB: 10 umol/L; DB: 2 umol/L; SGOT: 37 U/L; SGPT: 37 U/L; and SAP: 173 U/L). But after 9 months postoperatively, bilirubin level began to increase gradually. His investigations revealed TB 198 umol/L, DB 164 umol/L, SGOT 46 U/L, SGPT 33 U/L, and SAP 1176 U/L, and ultrasonography reported dilated intrahepatic bile ducts (IHBDs). He then underwent PTBD insertion for suspected HJ stricture (2013 March 03). However, his bilirubin level did not fall as expected, so he underwent re-positioning of the PTBD tube. Two weeks after this procedure, he presented with complaints of easy fatigability and passage of black tarry stool. His investigation revealed hemoglobin 7.4 gm/dl, platelet count 223000/uL PT/INR: 15 sec/1.07, TB 345 umol/L, DB 234 umol/L, SGOT 37 U/L, SGPT 22 U/L, and SAP 519 U/L. CT angiogram was performed to localize the source of bleeding, finding of which showed pseudoaneurysm of segment III hepatic artery of left lobe of liver (Figure 2).

With the diagnosis of hemobilia due to segment III hepatic artery (HA) pseudoaneurysm which likely occurred during PTBD manipulation, selective transarterial coil embolization was done, after which he improved symptomatically and his Hb normalized. He was continued on PTBD through which HJ stricture was dilated gradually with balloon catheters of size ranging from 6Fr to 12Fr. Following adequate dilation, his PTBD tube was removed. Since then, he has been under regular follow-up.

## 3. Discussion

One of the fundamental causes of BDI during cholecystectomy is the removal of gallbladder without proper dissection and identification of structures in Calot's triangle, before transecting cystic duct [5]. Magnetic resonance cholangiopancreatography can identify the site of injury, length of injured bile duct and variation in biliary tree anatomy with a diagnostic accuracy of 95% [6] to 100% [5]. BDI can range from minor bile leaks to major transection. There are several endoscopic techniques available for biliary drainage and decompression, e.g., biliary stent placement, biliary sphincterotomy, and nasobiliary drainage [7–9]. Endoscopic therapies aim at reducing transpapillary pressure gradient and improving transpapillary flow, which decreases the extravasation out of the biliary tract. This reduction in bile leakage allows healing of bile duct lesion injuries without direct surgical repair. However, if a major BDI occurs, i.e., a complete dissection of the common bile duct (CBD), surgical management is required [10]. The most common type of injury seen during cholecystectomy is Strasberg's E2 [6] which correlates with bismuth type II, which is classified as a major BDI.

The time period from cholecystectomy to referral to a higher center also determines the increase in risk of morbidities faced by BDI patients [11]. This might be due to higher rates of interventions and re-interventions these patients undergo before referral.

The timing of surgical repair of bile duct injury sustained during cholecystectomy influences the need for reoperation. Late repair beyond 45 days is usually recommended in view that people undergoing earlier repair had more risk of reoperation [12].

Hepaticojejunostomy is usually the preferred method of biliary reconstruction in patients with bile duct injury for more than 3 months. One of the most feared complications following hepaticojejunostomy is an anastomotic stricture with reported incidences reaching 2%-25% [13, 14]. This can present up to 17 to 20 years following surgery [14].

Hepatic arterial injury is a relatively rare but serious complication of PTBD occurring in 1.9%-2.5% of PTBD procedure [15, 16]. Benign disease and left-sided PTBD [15] has been identified as risk factors for HA injury. Hemobilia is the most common clinical indicator of HAI (79.2%-87%) [15, 16]. Substantial arterial bleeding occurring within 1 week after PTBD is primarily due to arteriocholedochal fistula and that occurring after 1 week is due to pseudoaneurysm [15]. In such a setting, transcatheter arterial embolization is a safe and effective method for treating hepatic arterial injury following PTBD [15, 17].

## 4. Conclusions

Bile duct injury during cholecystectomy can lead to a multitude of complications resulting in a “biliary cripple.” However, timely management can lead to improved quality of life. It is therefore prudent to identify cases with BDI as early as possible. We recommend intraoperative cholangiogram in cases with anatomical obscuration and/or variation as well as during the surgeon's learning curve.

## Data Availability

Data sharing not applicable to this article as no datasets were generated or analyzed during the current study.
